# *Moringa* (*Moringa oleifera*) Leaf Attenuates the High-Cholesterol Diet-Induced Adverse Events in Zebrafish: A 12-Week Dietary Intervention Resulted in an Anti-Obese Effect and Blood Lipid-Lowering Properties

**DOI:** 10.3390/ph18091336

**Published:** 2025-09-05

**Authors:** Kyung-Hyun Cho, Ashutosh Bahuguna, Yunki Lee, Ji-Eun Kim, Sang Hyuk Lee, Krismala Djayanti

**Affiliations:** Raydel HDL Research Institute, Medical Innovation Complex, Daegu 41061, Republic of Korea

**Keywords:** carboxymethyllysine, dyslipidemia, glucose, HMG-CoA reductase, paraoxonase, oxidative stress, obesity

## Abstract

**Objective:** The study investigates the dietary effects of *Moringa oleifera* leaf powder on obesity, blood biochemical parameters, and organ health in hyperlipidemic zebrafish (*Danio rerio*). **Methodology**: Adult hyperlipidemic zebrafish (n = 56/group) were fed for 12 weeks either with a high-cholesterol diet (HCD, 4% *w*/*w*) or HCD supplemented with 0.5% (*w*/*w*) *M. oleifera* leaf powder (0.5% MO) or HCD with 1.0% (*w*/*w*) *M. oleifera* leaf powder (1.0% MO). At different time points (0 to 12 weeks), the survivability and body weight (BW) of zebrafish were measured, while various biochemical and histological evaluations were performed after 12 weeks of feeding the respective diets. Additionally, an in silico approach was used to assess the binding interactions of MO phytoconstituents with 3-hydroxy-3-methylglutaryl coenzyme A (HMG-CoA) reductase. **Results**: Following 12-week supplementation, higher zebrafish survivability was observed in the MO-supplemented groups compared to the survivability of the HCD group. Relative to the initial BW, only 4% BW enhancement was observed post 12 weeks of dietary intake of 1.0% MO, in contrast to 27% BW gain in the HCD group. MO supplementation at both (0.5% and 1.0%) effectively mitigates the HCD-induced dyslipidemia and significantly minimizes the atherogenic coefficient and atherogenic index. Similarly, MO reduces elevated blood glucose levels, the ALT/AST ratio, and augments ferric ion reduction (FRA) and paraoxonase (PON) activity in a dose-dependent manner. Likewise, MO (particularly at 1.0%) effectively restrained HCD-induced steatosis, hepatic interleukin (IL)-6 production, and protected the kidneys, testes, and ovaries from oxidative stress and cellular senescence. The in silico findings underscore that the six phytoconstituents (chlorogenic acid, isoquercetin, kaempferol 3-*O*-rutinoside, astragalin, apigetrin, and myricetin) of MO exhibited a strong interaction with HMG-CoA reductase active and binding site residues via hydrogen and hydrophobic interactions. **Conclusions**: The findings demonstrated an antioxidant, anti-inflammatory, and hypoglycemic effect of MO, guiding the events to prevent HCD-induced metabolic stress and safeguard vital organs.

## 1. Introduction

The global prevalence of obesity has surged dramatically, evidenced by a 3-fold increase in the number of obese individuals between 1975 to 2016 [1]. Obesity is among the key instigators for metabolic syndrome, including insulin resistance, type II diabetes, fatty liver, hypertension, dyslipidemia, and cardiovascular diseases [2]. Apart from the genetic cause, lack of physical activity, intake of high-calorie diet, insufficient sleep, stress, and various kinds of drugs are the chief causes of obesity [3] and dyslipidemia [4,5]. Therefore, altering lifestyle and food habits are the first steps to managing obesity and dyslipidemia; however, these modifications sometimes prove insufficient and require the intervention of a pharmacological approach.

There is a range of synthetic drugs available to curb obesity [1] and dyslipidemia [6]; however, the side effects associated with their prolonged use are often a matter of concern [1,7]. Drugs primarily belonging to the statin class are available to treat dyslipidemia [6] by targeting 3-hydroxy-3-methylglutaryl coenzyme A (HMG-CoA) reductase, a rate-limiting enzyme in cholesterol biosynthesis [7]. Contrary to synthetic drugs, various plants and herbs and their metabolites, including a variety of polyphenols, alkaloids, saponins, and terpenes, have been documented for their positive impact on obesity and dyslipidemia [7,8,9,10] with little to no side effects [7], thus providing an alternative to treat such metabolic disorders.

Among the herbal medicines and functional foods, *Moringa oleifera* has caught substantial attention due to its high nutritional value and distinct pharmacological properties [11]. Nearly all parts of *M. oleifera* (bark, flower, leaves, seeds, fruits, and root) have nutritional and medicinal virtues and are traditionally used to treat a variety of ailments, including skin infections, anxiety, asthma, anemia, joint pain, blood pressure issues, respiratory disorders, and diabetes [12]. In particular, *M. oleifera* leaves are a rich source of minerals (such as iron, phosphorus, calcium, and potassium), proteins, essential amino acids, vitamins, and phenolic compounds, responsible for a variety of health-beneficial effects [9,12].

Despite studies conducted on *M. oleifera,* a comprehensive study deciphering the effect of *M. oleifera* leaf on the metabolic stress induced by a high-cholesterol diet (HCD) in zebrafish is scarce. Given this, the present study was designed to evaluate the effect of 12 weeks of dietary intake of *M. oleifera* leaf powder (MO) at 0.5% and 1.0% (*w*/*w*) on obesity, dyslipidemia, hyperglycemia, and oxidative stress induced by a high-cholesterol diet (HCD) in adult hyperlipidemic zebrafish (*Danio rerio*). Additionally, the effect of MO intake was evaluated on the adverse effects caused by HCD on the liver, kidney, and reproductive organs. In addition, the 3-hydroxy-3-methylglutaryl coenzyme A (HMG-CoA) reductase inhibitory effect of major phytoconstituents of MO was examined using the computational approach.

The zebrafish was selected as a model organism owing to its high genomic similarity and comparable organ development and function with humans [13]. Specifically, zebrafish and humans share critical aspects of lipid metabolism, including key receptors, lipoproteins, and enzymes involved in lipoprotein metabolism [14]. In addition, the high-calorie diet-induced obesity model of zebrafish correlated with human hepatic steatosis and displayed nearly similar changes in gene expression to mammalian obesity [15]. Moreover, HCD induces a variety of events in zebrafish that resemble human conditions, such as lipid accumulation, development of vascular lesions, lipoprotein oxidation, and macrophage lipid uptake [15], establishing zebrafish as a valuable in vivo preclinical model for metabolic disorders and human diseases.

## 2. Results

### 2.1. In Vitro Antioxidant Activity

**Figure 1:**
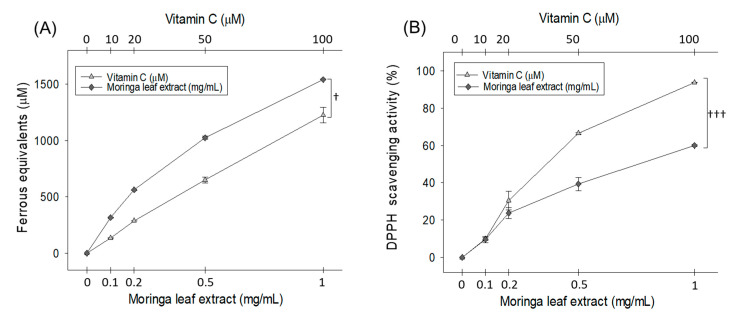
In vitro antioxidant activity of *Moringa oleifera* leaf extract (MOE). (**A**) Ferric ion reduction ability (FRA), (**B**) DPPH free radical scavenging activity. Data points represent the mean ± SD of triplicate experiments. The ^†^ (*p* < 0.05) and ^†††^ (*p* < 0.001) highlight the statistical significance between the moringa and vitamin C groups using a *t*-test.

The antioxidant potential of *M. oleifera* leaf extract (MOE) was determined using DPPH free radical scavenging and the ferric ion reduction (FRA) assays (Figure 1). In the FRA assay, MOE (0.1, 0.2, 0.5, and 1 mg/mL) exhibited dose-dependent ferric ion reduction activity, corresponding to 315 ± 5, 562 ± 5, 1024 ± 13, and 1540 ± 5 μM ferrous ion equivalent, respectively. Notably, at 1 mg/mL, MOE demonstrated superior FRA activity compared to the 100 μM of vitamin C (positive control). Likewise, DPPH free radical scavenging activity increased in a dose-dependent manner, reaching 9.6%, 23.7%, 39.3%, and 60.1% at 0.1, 0.2, 0.5, and 1 mg/mL MOE, respectively.

### 2.2. Moringa Rescues Zebrafish Embryos from the CML-Induced Oxidative Stress

**Figure 2:**
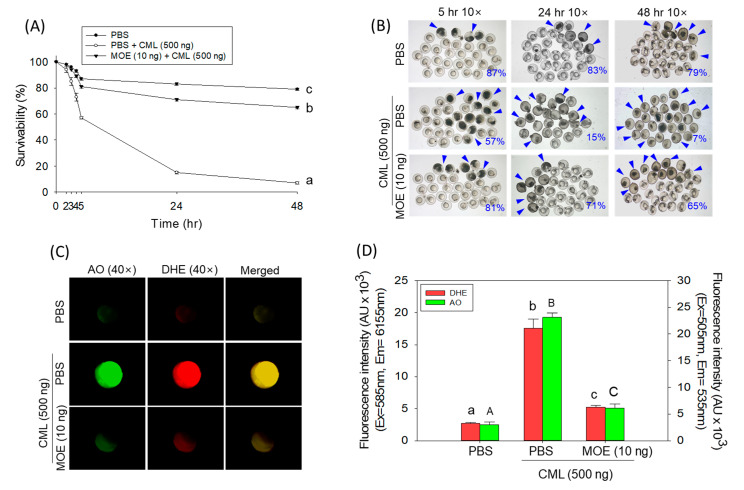
Effect of *Moringa oleifera* leaf extract (MOE) on the survivability of zebrafish embryos (n = 100) against the carboxymethyllysine (CML) posed toxicity. (**A**) Embryo survivability during 48 h post injection. (**B**) Pictorial view of the embryos. The blue arrow highlights the dead embryos. The numerical value inside the images depicts the percentage of embryo survivability. (**C**) Dihydroethidium (DHE) and acridine orange (AO) fluorescent imaging. (**D**) Quantification of DHE and AO fluorescent intensities employing Image J software (version 1.53, https://imagej.net/ij, accessed on 6 June 2023). Alphabets (a–c and A–C) represent the statistical significance between the groups using one-way ANOVA following Tukey post hoc analysis at *p* < 0.05.

CML exhibited pronounced embryotoxicity effects, as indicated by a steep decline in embryo survivability from 98% (at 2 h post injection) to 57%, 15%, and 7% at 5, 24, and 48 h, respectively (Figure 2A,B). In contrast, embryos injected with PBS (control) maintain a high survivability of 79% at 48 h post injection. Co-administration of MOE (CML + MOE group) significantly (*p* < 0.05) improved embryo survivability to 71% and 65% at 24 and 48 h post injection, representing 4.7-fold and 9.1-fold increases in survivability than the CML-injected group at the respective time points, thereby highlighting the moringa protective effect towards CML-induced embryotoxicity.

In the CML-administered group, the maximum DHE (corresponding to ROS) and AO (indicating apoptosis) fluorescent intensities were observed that were 6.5-fold and 7.9-fold higher than the respective fluorescent intensities in the PBS-injected group (Figure 2C,D). In the CML + MOE group, significantly 3.3-fold and 3.7-fold reduced DHE and AO fluorescent intensities were quantified with respect to the CML-injected group. The results confirm the cellular antioxidant and anti-apoptotic activity of moringa against the external stress posed by the CML.

### 2.3. Effect of Moringa on Hyperlipidemic Zebrafish Survivability and Body Weight

**Figure 3:**
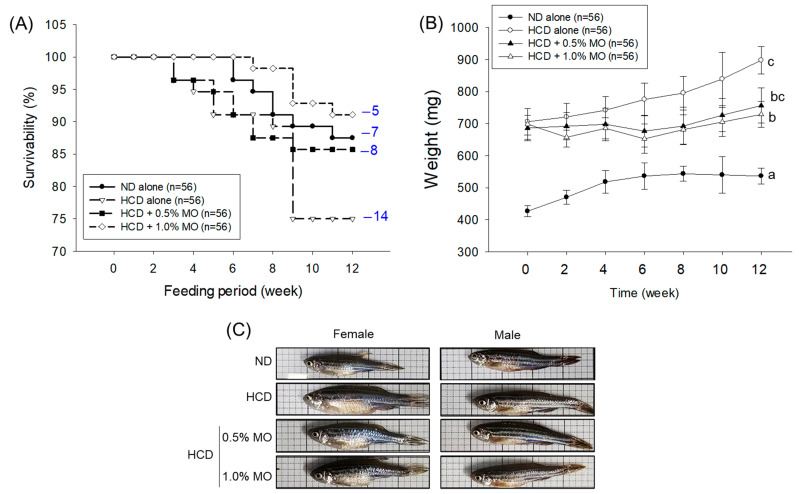
Zebrafish (n = 56) (**A**) survivability and (**B**) body weight (BW) changes during 12 weeks of feeding of different diets. The numerical value in blue font highlights the number of dead zebrafish in the respective group. (**C**) Pictorial view (representative images) of zebrafish among the groups after 12 weeks of feeding. ND: normal diet; HCD: high-cholesterol diet; HCD + 0.5% MO or 1.0% MO: high-cholesterol diet infused with 0.5% or 1.0% *Moringa oleifera* leaf, respectively. Alphabets (a–c) represent the statistical significance between the groups using one-way ANOVA following Tukey post hoc analysis at *p* < 0.05.

During the first 2 weeks of feeding, zebrafish exhibited 100% survivability across the experimental groups (Figure 3A). However, in the HCD group, survivability decreased to 94% by week 3 and further declined to 75% by week 9, remaining stagnant thereafter until week 12 of feeding (Figure 3A). Contrary to this, improved zebrafish survivability was observed in the HCD co-supplemented with *M. oleifera* leaf powder (MO 0.5% and 1.0% groups). Specifically, the 1.0% MO-supplemented group maintained 100% survivability until week 6, with a slight decline to 91.1% in week 12. Compared to the HCD group, the 0.5% and 1.0% MO-supplemented groups showed 12.3% and 21.5% higher survivability, respectively, demonstrating a dose-dependent effect of MO against HCD-induced mortality.

The baseline (week 0) body weight (BW) of all the HCD pre-fed groups (i.e., HCD, HCD + 0.5% MO, and 1.0% MO) was substantially higher by ~1.6-fold than the average BW of the ND-fed group, documenting the successful implementation of HCD in elevating BW. During 12 weeks of feeding, a periodical enhancement in BW was observed in the HCD group. At the final 12 weeks of feeding, a 27% enhancement of the BW (898.8 ± 43.7 mg) was observed in the HCD group compared to the week 0 BW (706.1 ± 42.5 mg). Juxtaposed, the co-supplementation of 0.5% and 1.0% MO showed merely a 10% and 4% enhancement in the BW, respectively, after 12 weeks compared to their respective BW at week 0. When compared to the final day (week 12) BW, 0.5% (757.5 ± 55.2 mg) and 1.0% (728.6 ± 40.3 mg) MO-supplemented groups exhibited a notable 16% and 19% reduction in BW than the BW of the HCD-supplemented group (898.8 ± 43.7 mg), highlighting the dose-dependent effect of MO towards the mitigation of HCD-derived BW gain.

At different time points (0, 6, and 12 weeks), a similar food consumption rate (~95–100%) was observed across the groups, indicating a non-significant effect of the different dietary formulations on the liking/disliking and appetite of zebrafish.

### 2.4. Moringa Positively Modulates the Plasma Lipid Profile of Hyperlipidemic Zebrafish

**Figure 4:**
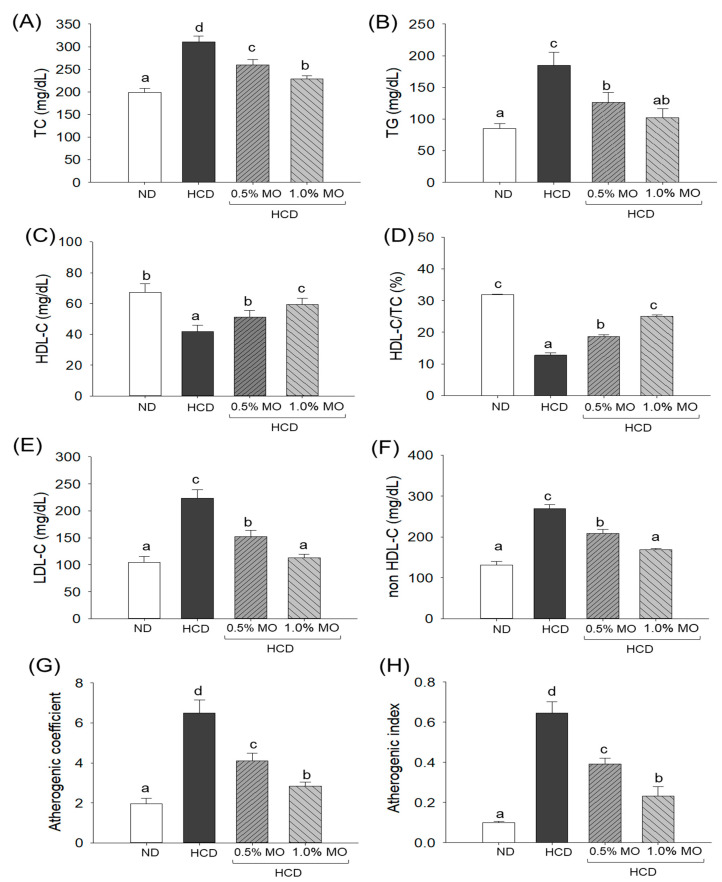
Plasma lipid profile of the zebrafish following 12 weeks of feeding of the respective diets. (**A**) Total cholesterol (TC), (**B**) triglycerides (TG), (**C**) high-density lipoprotein cholesterol (HDL-C), (**D**) percentage ratio of HDL-C/TC, (**E**) low-density lipoprotein cholesterol (LDL-C), (**F**) non-HDL-C, (**G**,**H**) atherogenic coefficient and atherogenic index, respectively. ND: normal diet; HCD: high-cholesterol diet; HCD + 0.5% MO or 1.0% MO: high-cholesterol diet infused with 0.5% or 1.0% *Moringa oleifera* leaf, respectively. Alphabets (a–d) above the bar graphs represent the statistical significance between the groups, as determined by one-way ANOVA followed by Tukey’s post hoc analysis at *p* < 0.05.

The highest level of TC (310.9 ± 12.6 mg/dL), TG (184.8 ± 20.3 mg/dL), LDL-C (223.5 ± 15.8 mg/dL), and non-HDL-C (269.1 ± 10.6 mg/dL) was observed in the HCD consumed groups that were significantly (*p* < 0.05) 1.6-fold, 2.2-fold, 2.1-fold, and 2.0-fold higher than their respective levels in the ND control group (Figure 4). The supplementation of 0.5% and 1.0% MO effectively reduced the HCD-elevated levels of TC by 16.4% (259.8 ± 12.1 mg/dL) and 26.6% (228.3 ± 6.9 mg/dL), TG by 31.5% (126.6 ± 15.3 mg/dL) and 44.6% (102.3 ± 13.8 mg/dL), LDL-C by 31.1% (151.9 ± 11.8 mg/dL) and 49.5% (112.8 ± 6.9 mg/dL), and non-LDL-C by 22.5% (208.6 ± 9.8 mg/dL) and 37.3% (168.7 ± 3.6 mg/dL), respectively.

In contrast to TC, TG, and LDL-C, the lowest HDL-C (41.8 ± 4.0 mg/dL) level was observed in the HCD group, which was significantly elevated by 42.3% following the supplementation of 1.0% MO (59.5 ± 4.1 mg/dL) (Figure 4C). Unlike 1.0% MO, 0.5% MO supplementation displayed a non-significant (*p* > 0.05) effect on the improvement of HCD-disturbed HDL-C levels. However, both 0.5% and 1.0% MO supplementation significantly augmented the HCD-diminished HDL-C/TC ratio (12.7%) by 1.5-fold (*p* < 0.05) and 2.1-fold (*p* < 0.05), respectively (Figure 4D). In addition, an elevated atherogenic coefficient (6.5) and atherogenic index (0.64) were noticed in the HCD-consumed group, which were significantly reduced to 36.9% and 39.3% by the supplementation of 0.5% MO and 56.3% and 64.2%, respectively, by the supplementation of 1.0% MO (Figure 4G,H).

### 2.5. Quantification of Blood Glucose, Antioxidants, and Hepatic Function Biomarkers

**Figure 5:**
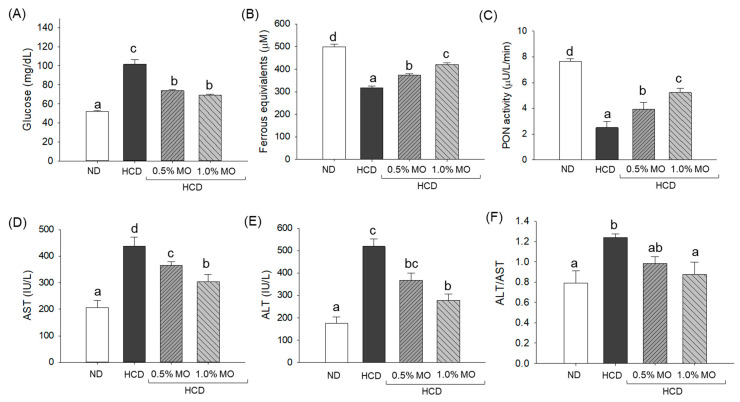
Quantification of (**A**) glucose, (**B**) ferric ion reduction ability (FRA), (**C**) paraoxonase (PON) activity, (**D**) aspartate aminotransferase (AST), (**E**) alanine aminotransferase (ALT), and (**F**) ratio of ALT/AST of plasma from the zebrafish following 12 weeks of feeding of the respective diets. ND: normal diet; HCD: high-cholesterol diet; HCD + 0.5% MO or 1.0% MO: high-cholesterol diet infused with 0.5% or 1.0% *Moringa oleifera* leaf, respectively. Alphabets (a–d) above the bar graphs represent the statistical significance between the groups, as determined by one-way ANOVA followed by Tukey’s post hoc analysis at *p* < 0.05.

Compared to the blood glucose of the ND control group (51.7 ± 0.91 mg/dL), an ~2-fold elevated blood glucose level was observed in the HCD (101.5 ± 5.1 mg/dL) group which was substantially 27.3% (73.8 ± 1.1 mg/dL) and 31.7% (69.3 ± 0.7 mg/dL) reduced following the co-supplementation of 0.5% and 1.0% MO, respectively (Figure 5A).

As depicted in Figure 5B,C, a substantially reduced FRA (318.2 ± 7.1 μM) and PON (2.5 ± 0.5 μU/L/min) activity was quantified in the blood of HCD-consumed zebrafish compared to the FRA (499.6 ± 10.8 μM) and PON (7.6 ± 0.21 μU/L/min) activity of the ND group. Supplementation of MO at 0.5% and 1.0% elevated the HCD-diminished PON activity by 1.6-fold and 2.1-fold, respectively. Likewise, a significant 16.9% and 32.1% higher FRA activity was observed in the 0.5% MO and 1.0% MO-supplemented groups, respectively, compared to the HCD-supplemented groups.

A notable 2.2-fold and 2.9-fold elevation was observed in the AST and ALT levels of the HCD consumed group compared to the ND (control) group (Figure 5D,E). The supplementation of MO at 0.5% and 1.0% effectively prevents the HCD-induced elevation of AST levels by 16.7% and 30.7%, and ALT levels by 29.3% and 46.4%, respectively. Additionally, the least ALT/AST ratio was observed in the ND group, which was significantly increased by 37.2% (*p* < 0.05) following the consumption of HCD (Figure 5F). The supplementation of 1.0% MO effectively lowered the ALT/AST ratio, evident by a significant 22.8% (*p* < 0.05) reduction in ALT/AST value in the 1.0% MO-supplemented group than the HCD-consumed group. The results signify the effective role of MO in preventing the elevation of HCD-induced plasma AST and ALT and restoration of glucose and antioxidant variables.

### 2.6. Moringa Protects Against the High-Cholesterol Diet-Triggered Liver Damage of Zebrafish

**Figure 6:**
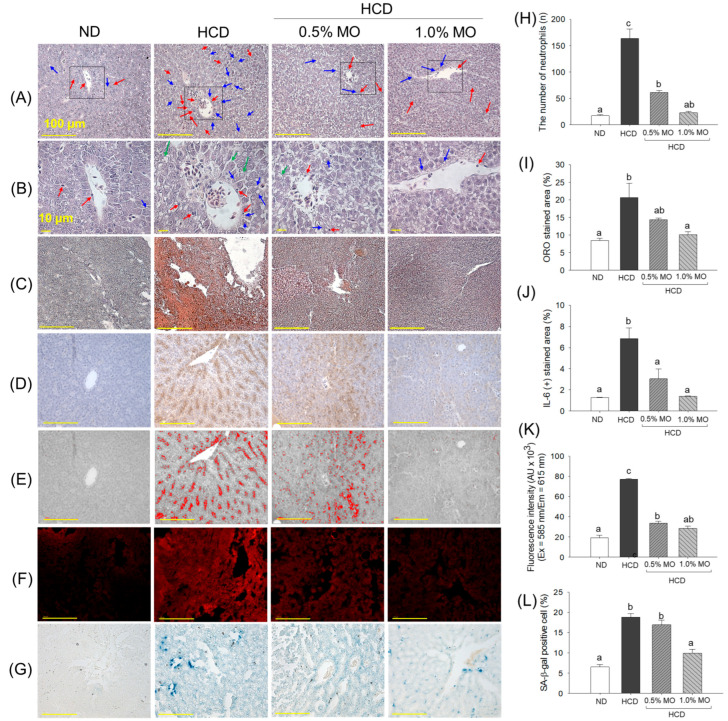
Liver histology of the zebrafish following 12 weeks of feeding of the respective diets. (**A**) Hematoxylin & eosin staining (H&E), (**B**) magnified view of the H&E-stained area covered by the black box in (**A**); red and blue arrows indicate neutrophils and lipid droplets, respectively, while the green arrow highlights the swollen hepatocyte. (**C**) Oil red O (ORO) staining, (**D**) immunohistology (IHC) for the detection of interleukin (IL)-6, (**E**) red conversion of the IHC-stained area using ImageJ software at brown color threshold value (20–120), red conversion was performed to enhance the clarity of the IHC-stained area, (**F**) dihydroethidium (DHE) fluorescent staining, and (**G**) senescent-associated β-galactosidase (SA-β-gal) staining; scale bar, 100 μm. Quantification of (**H**) neutrophils, (**I**) ORO-stained area, (**J**) IL-6-stained area, (**K**) DHE fluorescent intensity, and (**L**) SA-β-gal-positive cells. ND: normal diet; HCD: high-cholesterol diet; HCD + 0.5% MO or 1.0% MO: high-cholesterol diet infused with 0.5% or 1.0% *Moringa oleifera* leaf, respectively. Alphabets (a–c) above the bar graphs represent the statistical significance between the groups, as determined by one-way ANOVA followed by Tukey’s post hoc analysis at *p* < 0.05.

The liver H&E staining revealed the highest neutrophil (indicated by blue arrow) and lipid accumulation (indicated by red arrow) in the hepatic tissue from the HCD group (Figure 6A,B,H). When compared with the ND group, a 2-fold higher prevalence of neutrophils was observed in the HCD group. The supplementation of MO at 0.5% and 1.0% effectively reduced the neutrophil counts by 2.6-fold and 7.2-fold, respectively, compared to the HCD-consumed group. Consistent with the H&E findings, the ORO staining revealed a high accumulation of lipids in the liver of the HCD-consumed group (Figure 6C,I). The HCD-induced lipid accumulation in the liver was effectively prevented by the co-supplementation of 1.0% MO, as reflected by a significantly 2.1-fold-reduced ORO-stained area in the 1.0% MO-supplemented group compared to the ORO-stained area of the HCD-consumed group. Surprisingly, a non-significant effect (*p* > 0.05) at 0.5% MO was observed on the HCD-induced fatty liver changes.

The IHC staining revealed a significantly 5.2-fold higher IL-6 production in the hepatic tissue from the HCD group compared to the IL-6 level detected in the ND group (Figure 6D,E,J). The HCD-provoked IL-6 production was significantly 2.2-fold and 4.8-fold reduced following the co-supplementation of 0.5% and 1.0% MO, respectively.

The DHE (Figure 6F,K) and SA-β-gal staining (Figure 6G,L) revealed massive ROS production and higher cellular senescence in the HCD-consumed groups, which were significantly reduced by 63.1% and 47.3%, respectively, following the supplementation of 1.0% MO. Additionally, MO at 0.5% effectively reduced HCD-induced ROS generation, as evidenced by a 56.1% decrease in DHE fluorescent intensity compared to the HCD group. However, a non-significant effect of 0.5% MO supplementation was observed against the HCD-induced cellular senescence.

### 2.7. Moringa Prevents High-Cholesterol Diet-Induced Kidney Damage, Ros Generation, and Cellular Senescence

**Figure 7:**
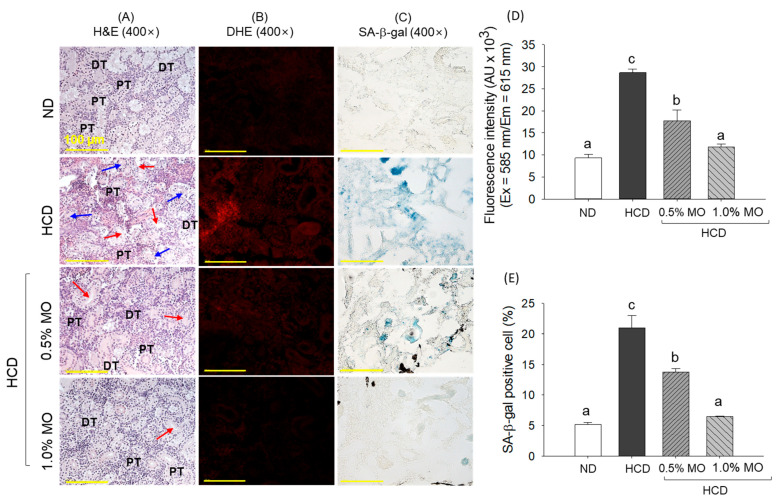
Kidney histology of the zebrafish following 12 weeks of feeding of the respective diets. (**A**) Hematoxylin & eosin staining (H&E). DT and PT are abbreviated for distal tubules and proximal tubules; the red arrow indicates elevated tubular lumen while the blue arrow indicates cellular debris in the tubular lumen. (**B**) Dihydroethidium (DHE) fluorescent staining, and (**C**) Senescent-associated β-galactosidase (SA-β-gal) staining; scale bar, 100 μm. Quantification of (**D**) DHE fluorescent intensity and (**E**) SA-β-gal-positive cells. ND: normal diet; HCD: high-cholesterol diet; HCD + 0.5% MO or 1.0% MO: high-cholesterol diet infused with 0.5% or 1.0% *Moringa oleifera* leaf, respectively. Alphabets (a–c) above the bar graphs represent the statistical significance between the groups, as determined by one-way ANOVA followed by Tukey’s post hoc analysis at *p* < 0.05.

The H&E staining of the HCD group revealed a disturbed cellular structure with unorganized distal (DT) and proximal tubules (PT), with a marked presence of elevated tubular lumen (indicated by red arrow) and cellular debris (indicated by blue arrow) in the tubular lumen (Figure 7A). In contrast, a well-organized tubular structure and arrangement, almost free from luminal debris, were observed in the ND (control) group. The HCD-induced kidney damage was substantially protected by the 0.5% and 1.0% MO supplementation; however, an occasional presence of dilated tubular lumen was noticed in the 0.5% MO-supplemented group.

The DHE staining showed the highest ROS level in the HCD group, which was significantly 3-fold higher than the ND group (Figure 7B,D). The co-supplementation of MO with HCD effectively prevents ROS generation, as depicted by the 1.6-fold and 2.4-fold less DHE fluorescent intensity in the 0.5% and 1.0% MO-supplemented groups than the DHE fluorescent intensity quantified in the HCD group. Likewise, higher senescent-positive cells (20.9%) were observed in the HCD group, which were significantly (*p* < 0.05) reduced to 13.8% and 6.4% by the co-supplementation of 0.5% and 1.0% MO, describing the dose-dependent potential of MO to contain the HCD-induced senescence (Figure 7C,E).

### 2.8. Moringa Prevents High-Cholesterol Diet-Induced Testes Damage

**Figure 8:**
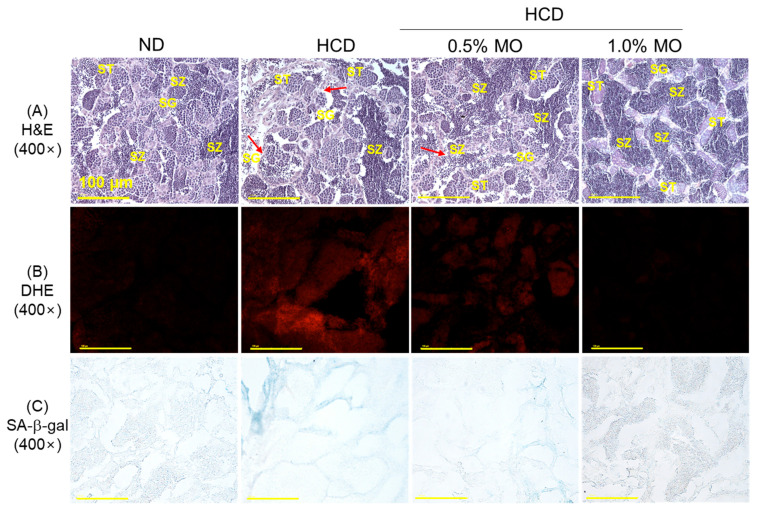

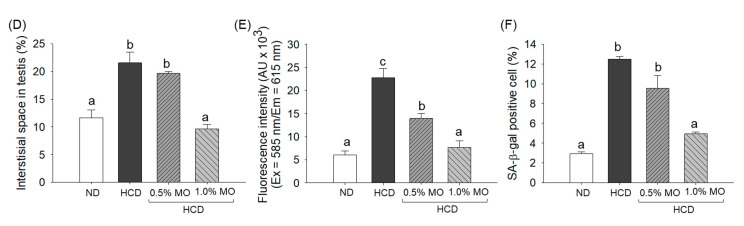
Testes histology of the zebrafish following 12 weeks of feeding of the respective diets. (**A**) Hematoxylin & eosin staining (H&E), SG, ST, and SZ are abbreviated for spermatogonia, spermatocytes, and spermatozoa, respectively. Red arrow indicates disruption of the basal lamina membrane. (**B**) Dihydroethidium (DHE) fluorescent staining, and (**C**) senescent-associated β-galactosidase (SA-β-gal) staining; scale bar, 100 μm. Quantification of (**D**) interstitial space between seminiferous tubules, (**E**) DHE fluorescent intensity, and (**F**) SA-β-gal-positive cells. ND: normal diet; HCD: high-cholesterol diet; HCD + 0.5% MO or 1.0% MO: high-cholesterol diet infused with 0.5% or 1.0% *Moringa oleifera* leaf, respectively. Alphabets (a–c) above the bar graphs represent the statistical significance between the groups, as determined by one-way ANOVA followed by Tukey’s post hoc analysis at *p* < 0.05.

Figure 8A,D, depicts the H&E staining images of the testes, which reveals the loosely arranged seminiferous tubules containing an irregular distribution of spermatogonia (SG), spermatocytes (ST), and spermatozoa (SZ). Also, a broadened interstitial space (21.5%) between the seminiferous tubules and occasional disruption of the basal lamina membrane (indicated by the red arrow) were noticed in the HCD-consumed group. In contrast, MO supplementation, mainly 1.0%, effectively prevented the HCD-induced testes damage reflected by the normal testicular cellular arrangement with significantly 2.2-fold lower interstitial space (9.6%) between the seminiferous tubules compared to the only HCD-supplemented group.

The DHE fluorescent (Figure 8B,E) and senescent staining (Figure 8C,F) revealed that the co-supplementation of 1.0% MO effectively prevented the HCD-induced ROS generation and cellular senescence in the testes. A significant 2.9-fold reduced DHE fluorescent intensity and 3.2-fold lowered senescent-positive cells in the HCD co-supplemented with 1.0% MO group than the exclusively HCD-consumed group, attesting to the effectiveness of MO to prevent the HCD-induced ROS and senescence in the testes. Notably, a non-significant effect of the MO at 0.5% was observed to inhibit the HCD-induced changes in the testes.

### 2.9. Moringa Prevents Ovary Damage of a High-Cholesterol Diet-Consuming Zebrafish

**Figure 9:**
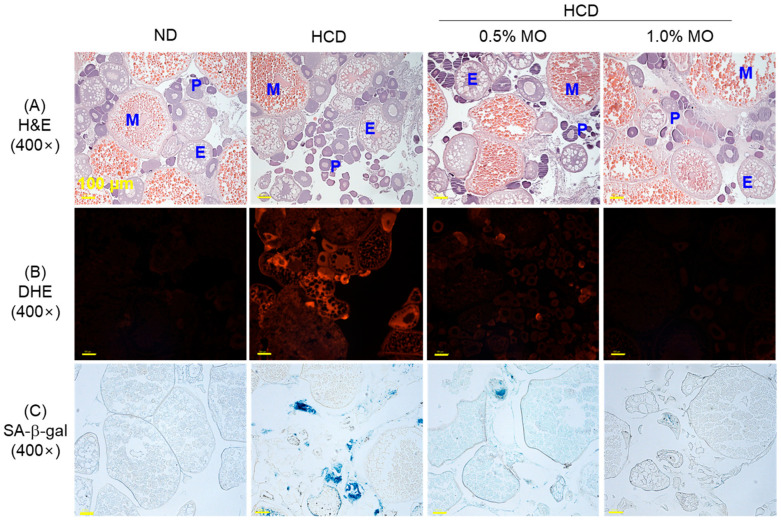

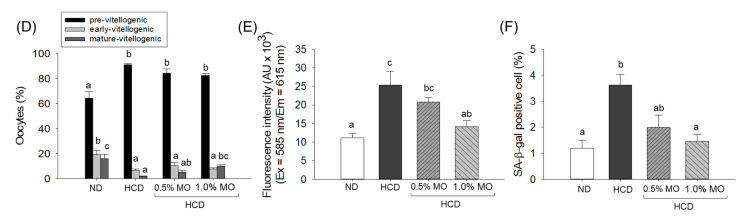
Ovary histology of the zebrafish following 12 weeks of feeding of the respective diets. (**A**) Hematoxylin & eosin staining (H&E). P, E, and M abbreviated for pre-, early, and mature vitellogenic oocytes. (**B**) Dihydroethidium (DHE) fluorescent staining and (**C**) senescent-associated β-galactosidase (SA-β-gal) staining; scale bar, 100 μm. Quantification of (**D**) oocyte counts, (**E**) DHE fluorescent intensity, and (**F**) SA-β-gal-positive cells. ND: normal diet; HCD: high-cholesterol diet; HCD + 0.5% MO or 1.0% MO: high-cholesterol diet infused with 0.5% or 1.0% *Moringa oleifera* leaf, respectively. Alphabets (a–c) above the bar graphs represent the statistical significance between the groups, as determined by one-way ANOVA followed by Tukey’s post hoc analysis at *p* < 0.05.

Figure 9A,D illustrates the significant impact of HCD on oocyte development, marked by the higher prevalence of previtellogenic oocytes (91.2%) and reduced numbers of early (6.9%) and mature oocytes (1.8%) compared to the ND group. Supplementation with the 0.5% and 1.0% MO exhibited no significant effect on HCD-induced alterations in the previtellogenic and early vitellogenic oocyte counts; however, the 1.0% MO-supplemented group demonstrated a notable 5.5-fold (*p* < 0.05) increase in mature oocytes (9.9%) relative to the HCD group (1.8%).

The highest DHE fluorescent intensity (Figure 9B,E) and senescence (Figure 9C,F) were observed in the HCD-consumed group, which was significantly reduced by 44.1% and 59.6%, respectively, following the co-supplementation of 1.0% MO. Unlike the 1.0% MO, supplementation with 0.5% MO had a non-significant effect on cellular senescence; however, it had a significant impact in reducing HCD-induced ROS production by 18.2%.

### 2.10. Molecular Docking

**Figure 10:**
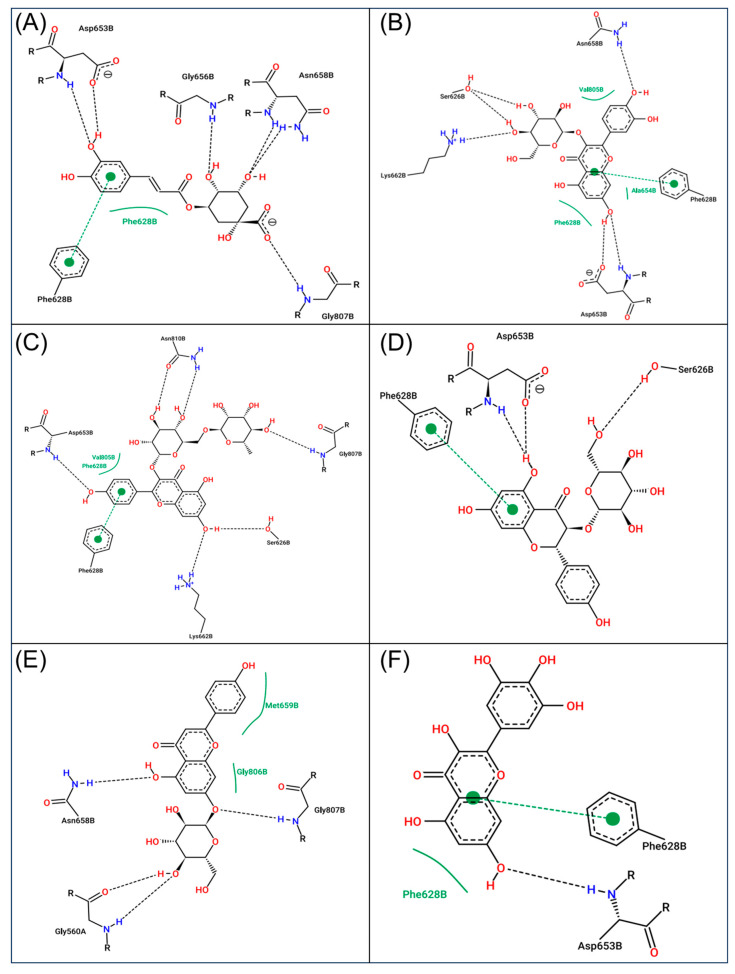
Two-dimensional interaction maps of six phytochemicals: (**A**) chlorogenic acid, (**B**) isoquercetin, (**C**) kaempferol 3-*O*-rutinoside, (**D**) astragalin, (**E**) apigetrin, and (**F**) myricetin of *Moringa oleifera* leaf with 3-hydroxy-3-methylglutaryl coenzyme A (HMG-CoA) reductase. The dotted black lines depict hydrogen bonding, while the green dotted line highlights the π-cation interaction between the ligand and receptor.

The molecular docking results predicted substantial binding affinities (−3.19 to −10.33 kcal/mol) of compounds from MO with the binding pocket of the target protein (HMG-CoA reductase) (Supplementary Table S1). Based on the highest binding affinity scores, six potential compounds (Table 1) were selected and analyzed for their interaction with the residues of HMG-CoA reductase. The 2D interaction maps (Figure 10A–F) revealed that chlorogenic acid, isoquercetin, kaempferol 3-*O*-rutinoside, astragalin, apigetrin, and myricetin-docked complexes were stabilized by the formation of 6, 6, 6, 3, 4, and 1 hydrogen bonds, respectively, with the active residues of HMG-CoA reductase (Figure 10). Additionally, these docked complexes were predicted for a considerable number of hydrophobic interactions with the receptor proteins (Table 1).

## 3. Discussion

*M. oleifera* is a “magical tree” gaining huge attention in the sector of food, medicine, and cosmetics due to its nutritional and therapeutic values [16]. Nearly all parts of *M*. *oleifera* harbor beneficial properties [16]. In particular, leaves are enriched with minerals, vitamins, and a variety of bioactive compounds (particularly phenolics and flavonoids) [16,17], which are largely responsible for the biological activities, making moringa leaves suitable for both therapeutic and commercial perspectives [16]. Due to these reasons, we have chosen moringa leaves over other parts to examine their in vitro, in silico, and in vivo efficacy against the adversity imposed by the exposure to CML and HCD.

The first protective effect of moringa against CML-induced events in zebrafish embryos was examined. CML is well known to generate the ROS/oxidative stress and inflammation leads to the induction of cell death [18,19,20]. Similarly, we have observed a high level of ROS generation and apoptotic cell death in zebrafish embryos injected with CML. A co-treatment of moringa leaf extract effectively suppressed the CML-induced ROS generation and apoptosis, leading to higher embryo survivability. CML accumulation has been reported in the adipose tissue of obese individuals, which triggers various harmful events, including obesity-induced inflammation and insulin resistance [21]. The current in vivo study demonstrated the effective role of moringa in mitigating CML-induced adverse events in zebrafish embryos, thereby raising the possibility that moringa may be effective in preventing CML-provoked adverse events in obesity. However, further study is required to verify this assumption.

The antioxidant activity of moringa, as observed in the present study (DPPH and FRA assay) and that mentioned in the literature [22], is the key defensive event behind the protective effect against the CML-triggered toxicity in embryos. The outcomes are supported by earlier reports documenting the effective cytoprotective role of moringa against oxidative insults due to its antioxidant nature, which directly scavenges free radicals and modulates cellular antioxidants via the nuclear factor erythroid 2-related factor 2 (Nrf2) nuclear transition, and has an anti-apoptotic effect by regulating caspase-mediated cell death [23].

Furthermore, the study was extended to examine the effect of dietary intervention of MO against the metabolic stress posed by the consumption of HCD. The intake of high cholesterol is associated with obesity [24], dyslipidemia [25], which in turn leads to insulin resistance [26], and adverse effects on vital organs. Herein, we have observed a substantial BW elevation in response to high cholesterol consumption, which is effectively prevented by the supplementation of moringa leaf, particularly when consumed at a 1.0% amount. Notably, a similar food consumption tendency was observed among the groups, underscoring that moringa does not affect food intake or appetite. These results are supported by previous findings documenting the effective role of moringa in combating high-fat diet-induced obesity in rats [8]. Additionally, studies have documented the effect of moringa on adipogenesis by regulating C/EBPα, adiponectin, FABP4, and PPARγ [9], as well as augmenting thermogenesis in adipose tissue, which testifies to the anti-obesity effect of moringa [9] and supports the current findings.

In addition to its inhibitory effect on BW elevation, moringa proved effective in counteracting HCD-induced dyslipidemia by reducing the HCD-elevated TC, TG, LDL-C, and non-HDL-C levels, while augmenting HDL-C levels. Of note, the HCD heightened atherogenic coefficient and index were substantially mitigated by moringa. Results align well with previous studies that deciphered the positive effect of moringa leaf towards mitigating dyslipidemia in albino rats and mice [9]. Compared to mice, zebrafish offer some distinct properties that closely mimic human lipid metabolism; for instance, zebrafish possess cholesteryl ester transfer protein (CETP), an essential component of human lipid metabolism that is absent in mice [14]. Also, the report suggested the effect of moringa on reducing cholesterol absorption by inhibiting pancreatic cholesterol esterase activity and cholesterol micellization formation [27] which leads to the attenuation of hypercholesterolemia and obesity [28,29]. Additionally, studies have explained the cholesterol-regulating effect of moringa by promoting bile acid synthesis from cholesterol and subsequent fecal removal in rats fed a high-fat and cholesterol diet [30]. Strikingly, some reports depict the effect of moringa leaf and its phytoconstituents on the activity inhibition of HMG-CoA-reductase activity [30,31,32], a main rate-limiting enzyme in cholesterol biosynthesis and the primary target of statins [33]. Regardless of the suppressive effect of moringa on the HMG-CoA-reductase activity, no studies so far have highlighted the interaction between the moringa phytoconstituents and HMG-CoA-reductase. Concerning this, molecular docking was performed in the present study using the critical phytoconstituent of moringa leaf, targeting HMG-CoA-reductase. The findings outlined a strong interaction between the moringa leaf phytoconstituents (chlorogenic acid, isoquercetin, kaempferol 3-*O*-rutinoside, astragalin, apigetrin, and myricetin) and the target protein (HMG-CoA-reductase), with a substantial docking score, suggesting the inhibitory potential of moringa towards HMG-CoA-reductase. Despite the substantial in silico interactions, a precise mechanism underlying the dyslipidemic effect of moringa co-supplementation warrants further investigation.

The elevated plasma FRA and PON activity are important markers reflecting a better antioxidant status. Precisely, PON is a vital enzyme associated with HDL, and its higher activity is responsible for imparting the antioxidant property to HDL [34]. The study outcome showed that moringa substantially improved HCD-compromised plasma FRA and PON activity, underscoring moringa’s potential to boost the antioxidant status. The current findings are in line with previous reports that decipher the positive effect of moringa on plasma FRA activity [35]. Nonetheless, a limited study deciphered the antioxidant potential of moringa in relation to its impact on PON activity. The combined results of the plasma lipid profile and antioxidant activities revealed that moringa not only enhanced the HDL-C level but also impacted the HDL functionality in terms of its antioxidant properties, resulting in several beneficial effects. The results are associated with the in vitro antioxidant activity of moringa, which helps to stimulate FRA and PON activities, and raises the HDL-C level, consequently improving the HCD-induced oxidative stress and dyslipidemia in obese zebrafish.

An adverse effect of high cholesterol has been implicated in fatty liver changes [36], as well as the augmented production of cytokines and chemokines [36] that induce a proinflammatory response, leading to adverse effects on liver health. Consistently, we have observed adverse effects of HCD consumption on the liver, marked by high neutrophil infiltration, fatty liver changes, elevated ROS, and IL-6 production. On the contrary, the supplementation of moringa effectively mitigates HCD-induced adverse effects in the liver. The visible inhibitory effect of moringa on HCD-induced obesity, dyslipidemia, and hyperglycemia are among the key events to prevent liver damage as fatty liver is strongly linked with such a metabolic disorder [37]. The findings are consistent with earlier studies demonstrating the hepatoprotective effect of moringa in obese mice [38]. Moringa has been documented to have a hepatoprotective effect on mammals (e.g., mice) and vertebrates (e.g., zebrafish), which are physiologically distinct in several aspects, highlighting its broad relevance in mitigating liver damage. Notably, zebrafish, despite their physiological differences from mice, offer an additional advantage for pharmacological evaluation due to their small size, which allows for testing a large population, as seen in the present study, with 56 zebrafish per group.

The inhibitory effect of moringa on ROS generation and IL-6 production substantially contributes to protecting the liver against HCD-induced damage, as oxidative stress and inflammation are the key drivers/instigators of hepatic injury [39]. These findings are well aligned with previous reports highlighting moringa’s antioxidant properties that suppress ROS production and prevent oxidative damage to the liver [40]. A noteworthy anti-inflammatory effect of moringa has been documented by modulating the nuclear translocation of NFκB [40], an important regulatory molecule responsible for the expression of various inflammatory mediators, which is a key mechanism underlying the diminished IL-6 production in the moringa-supplemented group. In addition, the marked reduction in neutrophil counts observed in the moringa-supplemented group corroborates the existing literature demonstrating moringa’s inhibitory effect on the production of IL-8 [41], an important chemokine that recruits neutrophils and mediates tissue damage. Heightened senescent-positive hepatocytes were observed in the HCD-consumed group, which was effectively prevented following the moringa supplementation. The antioxidant effect of moringa, which limits HCD-induced ROS generation, is the primary reason for inhibiting senescence, as oxidative stress has been recognized as a major contributor to cellular events that lead to cellular senescence [42,43].

Additionally, AST and ALT are essential biomarkers of hepatic function, and their higher serum levels indicate hepatic injury. Particularly, the elevated ALT/AST ratio gives a precise indication of fatty liver damage [44]. Herein, moringa supplementation demonstrated a substantial effect in mitigating HCD-elevated levels of AST, ALT, and the ALT/AST ratio, underscoring its hepatoprotective effect. The findings of the plasma hepatic function biomarkers corroborate the histological observations and validate moringa’s hepatoprotective nature against the adversity caused by the HCD.

Adverse effects of HCD have been described on the kidney and reproductive organs. In response to HCD consumption, we have observed elevated ROS levels and senescence in the kidney, which were effectively inhibited by moringa supplementation. We speculated that the potent antioxidant activity of moringa triggers important events that mitigate HCD-induced nephrotoxicity, as high cholesterol has been reported to impose an oxidative load on the kidney, which substantially leads to kidney impairment [45]. This notion aligns with previous studies that have deciphered the effect of moringa on enhancing cellular antioxidant activity in the kidney [12], resulting in a nephroprotective effect against oxidative insult [12]. Besides the effect of moringa on augmenting of HDL-C levels and HDL-associated PON activity, which supports a nephroprotective effect and better kidney health, studies have described compromised PON activity and HDL-C levels in chronic kidney disease [46].

Similarly, moringa displayed diminished ROS production, cellular senescence, and protection of testes and ovary cellular integrity, which were impaired by HCD. The effect of moringa has been documented to protect the testes [47] and ovaries [48] through distinct mechanisms, including antioxidant effects. In one such study, moringa impacted enzymatic antioxidants in the testes and improved testicular health altered by high-fat diet-induced toxicity [47]. In the kidneys, testes, and ovaries of the moringa-supplemented group, the least cellular senescence was observed, primarily attributed to the lowest ROS production in these groups, as ROS-induced oxidative stress is one of the main instigators of cellular senescence [42,43].

Despite several key similarities with humans, zebrafish also have some limitations, such as their poikilothermic nature (cold-blooded) [49], unlike humans, which are homeothermic (warm-blooded); thus, there is a difference in the regulation of metabolic rate in response to environmental conditions [50]. Furthermore, the pattern of adipose tissue development and distribution differs considerably between zebrafish and humans [49]. Notably, unlike humans, zebrafish do not harbor brown adipose tissue [50], which plays a pivotal role in combating obesity and obesity-related metabolic ailments [51]. Therefore, the therapeutic outcomes of the present work need to be verified prudently in context to the MO response in humans.

## 4. Materials and Methods

### 4.1. Chemical and Reagents

Paraoxon ethyl (Cat: 36186), 2-phenoxyethanol (Sigma P1126); 5-bromo-4-chloro-3-indolyl-β-D-galactopyranoside (X-gal, Cat: B54252), oil red O (Cat: O625), dihydroethidium (DHE, Cat:37291), and *N*-ε-carboxymethyllysine (Cat:14580) were sourced from Sigma-Aldrich (St. Louis, MO, USA). All the remaining chemicals and reagents were of highest purity and employed as received unless specified.

### 4.2. Plant Material and Extraction Method

A finely ground *Moringa oleifera* leaf powder (MO) was sourced from Havana, Cuba. The leaf powder was analyzed for the detection of food preservatives and microbial contamination by Korea Advanced Food Research Institute, Seoul, Republic of Korea, and for the quantification of its important phytoconstituent (kaempferol) by Korea Polymer Testing and Research Institute, Seoul, Republic of Korea. A certificate of analysis is provided in Supplementary Figures S1 and S2.

For the preparation of the alcoholic extract, 25 g of MO was suspended in 100 mL of ethanol and kept under rotating conditions (120 rpm). After 24 h, the content was filtered, and the filtrate was centrifuged; the supernatant was collected and processed for drying to obtain the dried MO extract.

For the in vitro assay (Section 4.3) and microinjection into zebrafish embryos (Section 4.5), an extract of MO leaf (MOE) was used. For feeding adult zebrafish (Section 4.7), a ground MO was used.

### 4.3. In Vitro Antioxidant Ability

Diphenyl-picrylhydrazyl (DPPH) and ferric ion reduction (FRA) assay were performed following the previously described method [52]. For the DPPH assay, 10 μL of MO leaf extract (dissolved in water) at varied concentrations (0.1–1.0 mg/mL) was mixed with 190 μL of DPPH solution (24 mg/L). Simultaneous control experiment devoid of MO leaf extract was also set up under the same experimental conditions. Following 15 min incubation at room temperature (RT), absorbance (Abs, 517 nm) was recorded, and results were documented as percentage DPPH scavenging activity using the following equation: [(Abs control − Abs test)/Abs control] × 100.

For the FRA assay, 20 μL MO leaf extract (dissolved in water) at a final concentration (0.1–1.0 mg/mL) was mixed with the FRA reagent [52]. After 60 min incubation at RT, absorbance 593 was recorded, and the results were expressed as ferrous equivalent (μm).

### 4.4. Culturing of Zebrafish and Embryo Production

A 25-week-aged zebrafish (wild type AB strain) was cultured in the water tank connected with the circulating water supply following the standard guidelines of the Committee of Animal Care and Use of Raydel Research Institute, Daegu, Republic of Korea (code and date of approval RRI-23-001, 27 July 2023). A 28 °C water temperature and 14 h light and 10 h dark photoperiod were maintained throughout the experimental period. Zebrafish were fed twice (~9 am and 6 pm) with normal tetrabit (ND, Tetrabit D49304, Melle, Germany).

For embryo production, male and female zebrafish (1:2) were kept in the breeding tank and segregated from each other using a physical divider. After 16 h separation, the divider was removed, allowing the male and female zebrafish to mate uninterruptedly. Post 30 min of mating, embryos were collected, rinsed with water, and preserved in embryo medium (3 g of sea salt dissolved in 0.1 mg/100 mL methylene blue solution).

### 4.5. Microinjection to Zebrafish Embryos

Embryos (~1 h post fertilization) were randomly allocated into three distinct cohorts (n = 100 per group). The embryos in the PBS group received 10 nL microinjections of phosphate-buffered saline (PBS, control), while the embryos in the CML group were microinjected with 500 ng CML dissolved in 10 nL PBS. Embryos in the CML + MOE groups were microinjected with 500 ng CML dissolved in 10 nL PBS containing 10 ng of MOE. To avoid bias, microinjection was applied at nearly the same position in the embryo yolk across the group using a microcapillary pipette equipped with a pneumatic pump (PV830; World Precision Instruments, Sarasota, FL, USA) and a magnetic manipulator (MM33; Kantec, Bensenville, Chicago, IL, USA). Periodically (0–48 h post injection), embryos were visualized under the microscope to examine the survivability.

### 4.6. Dihydroethidium (DHE) and Acridine Orange (AO) Staining

At 5 h post injection, embryos of the respective groups were processed for the DHE and AO fluorescent staining, following the earlier described method [20]. In brief, embryos (n = 10/group) were transferred into a 24-well plate and incubated with 500 μL of DHE (30 μM) and AO (5 μg/mL) solution for 30 min in the dark at RT. Afterward, the stained embryos were washed three times with PBS and visualized under a microscope at excitation (Ex) and emission (Em) wavelengths of 565 (Ex)/615 (Em) and 505 (Ex)/535 (Em) for the detection of DHE- and AO-fluorescent intensity, respectively.

### 4.7. Preparation of Different Diets, Zebrafish Feeding, and Food Consumption Efficacy

The normal tetrabit (ND) was supplemented with 4% cholesterol (*w*/*w*) to prepare the high-cholesterol diet (HCD). The HCD was further mixed with 0.5% (*w*/*w*) and 1.0% *(w*/*w*) *M. oleifera* leaf powder (MO) to make moringa-enriched dietary formulations, which were named as HCD + 0.5% MO and HCD + 1.0% MO, respectively.

Zebrafish (n = 224) were randomly allocated into 4 groups (n = 56/group) and fed either with ND, HCD, HCD + 0.5% MO, and HCD + 1.0% MO for 12 weeks. Zebrafish (n = 56) in each specified group were kept in the four distinct tanks (n = 14/tank × 4 = 56) and fed the specified diets of 10 mg/zebrafish twice a day (9 a.m. and 6 p.m.), i.e., a cumulative diet of 280 mg/tank/day. Importantly, zebrafish in the HCD, HCD + 0.5% MO, and 1.0% MO groups were pre-fed only with HCD for 15 weeks prior to starting the intake of their respective diets. The 15 weeks of pre-feeding of HCD were conducted to induce metabolic stress in the zebrafish.

The BW of the zebrafish among the different groups was determined gravimetrically at the beginning (week 0), followed by a 2-week interval until week 12. At the same time, the survivability of zebrafish was assessed daily until 12 weeks after consumption of different diets.

The food consumption efficacy among all groups was analyzed at weeks 0, 6, and 12 to examine the effect of dietary composition on the liking or disliking and appetite of zebrafish. The food consumption was assessed post 30 min exposure to the respective diets using the following formula: [(specified amount of the food − residual amount of food)/specified amount of the food] × 100.

### 4.8. Blood and Organ Collection

After 12 weeks of consumption of different diets, zebrafish across all the groups were fasted for ~14 h and then sacrificed using hypothermic shock to collect blood and different organs. The blood (~2–5 μL) from each zebrafish (n = 14/tank) of the specialized group was collected, pooled, and mixed with PBS-ethylenediaminetetraacetic acid (1 mM) in a 2:3 ratio and subsequently processed for centrifugation to collect the plasma. To minimize circadian and feeding-related fluctuations, zebrafish from all groups were euthanized on the same day at a nearly similar time range (9 a.m. to 10.30 a.m.).

Different organs (liver, kidney, testes, and ovaries) were obtained surgically by dissecting zebrafish under the microscope. The respective organs were preserved separately in 10% formalin.

### 4.9. Lipid Profile and Biochemical Analysis of Blood

Commercial kits, following the manufacturers’ guidelines, were used to detect total cholesterol (TC), triglycerides (TG), high-density lipoprotein cholesterol (HDL-C), aspartate aminotransferase (AST), and alanine aminotransferase (ALT). supplementary Method Section S1 describes a detailed methodology. The blood glucose level was quantified using a digital blood glucose meter (AccuCheck, Roche, Basel, Switzerland).

Blood ferric ion reduction ability (FRA) and paraoxonase (PON) activity were determined using the method described earlier [53]. A detailed procedure is outlined in the supplementary Method Section S2.

### 4.10. Histological Analysis, Immunohistochemical (IHC), Dihydroethidium (DHE), and Senescent Staining

A thin (7 μm) tissue slice of the respective organs was sectioned using a cryo-microtome (Leica CN1510S, Leica Biosystem, Nussloch, Germany) after fixing the tissue in the FSC22 clear solution (Leica). The tissue section was processed for the hematoxylin and eosin (H&E) staining following the earlier described method [54].

The fatty liver changes were assessed using the Oil Red O (ORO) staining method, as described earlier [20]. In brief, the liver tissue (7 μm) section was covered with 0.25 mL of ORO solution, following 5 min incubation at 60 °C, then the section was rinsed with isopropanol (60%) and examined under the microscope.

The IHC staining was performed to detect the interleukin (IL)-6 in the hepatic tissue following the described methodology [55]. In brief, tissue section (7 μm) was covered for 16 h with 200× diluted IL-6 specific antibody (ab9324, Abcam, London, UK) in a moist and cool (4 °C) atmosphere. Subsequently, the section was developed using the enzyme-tagged secondary antibody (1000 × diluted) employing the EnVison + System HRP-labeled polymer kit (Code K4001, Dako, Glostrup, Denmark). Furthermore, the developed IHC-stained area (brown color) was interchanged with the red color at brown at a color threshold value (20–120) utilizing ImageJ software (version 1.53, https://imagej.net/ij, accessed on 6 June 2023) to minimize the background color and enhance the visual appearance of the IL-6-stained area.

The DHE staining was performed following the methodology as described in Section 4.4. Senescence was detected by senescent associated-β-galactosidase (SA-β-gal) staining [56]. Briefly, tissue section (7 μm) was overnight stained with 0.1% X-gal (5-bromo-4-chloro-3-indolyl-β-D-galactopyranoside) solution and examined under the microscope for the detection of blue-colored SA-β-gal-positive cells.

### 4.11. In Silico Evaluation

In silico interaction was performed between the different phytoconstituents of the *M. oleifera* leaf with the HMG-CoA reductase.

#### 4.11.1. Preparation of Ligands and Receptor

A total of 25 important phytoconstituents from the moringa leaf (Supplementary Table S1) were selected from the literature [40,57,58,59], and their 3D structures were retrieved from the PubChem database (https://pubchem.ncbi.nlm.nih.gov, accessed on 21 July 2025) and saved in SDF file format. The 3D structure of the target protein (HMG-CoA reductase, ID: 1HWK) co-crystalized with atorvastatin was downloaded from the Protein Data Bank (PBD, https://www.rcsb.org).

#### 4.11.2. Molecular Docking

The molecular docking between the ligands (phytoconstituents of moringa leaf) and HMG-CoA reductase was performed using the Autodock 4.2 tool [60]. Prior to ligand and receptor interactions, 3D structures of the receptor were processed for the addition of the polar hydrogen atoms, while non-polar hydrogen atoms of the ligands were merged and subsequently treated with Gasteiger partial charges. Also, the native ligand and other ions or compounds added during crystallization were removed during the processing of the protein structure. For creating flexible ligand conformations, all torsion angles were set free during the docking experiment conducted by AutoDock Vina version 1.2.7 [61]. The docked poses (2D interaction maps) with the highest docking scores and least root mean square deviation values for the respective ligands were selected for the computation analysis using online web server Poseview (https://proteins.plus/help/poseview, accessed on 23 July 2025). Additionally, hydrophobic interactions stabilizing the docked complex were computed for the docked poses by using online web server VinaLigGen (http://github.com/raghvendra44/VinaLigGen, accessed on 23 July 2025).

### 4.12. Statistical Analysis

The data distribution was tested for normality before evaluating intergroup differences using one-way ANOVA, followed by post hoc analysis with Tukey’s test in the SPSS software package (version 29, Chicago, IL, USA). To establish the pairwise statistical significance between the groups (bivariate data), a two-tailed t-test was performed.

## 5. Conclusions

A 12-week dietary intervention of moringa leaf exhibited a strong anti-obesity effect and countered HCD-induced dyslipidemia. In addition, moringa improved the plasma antioxidant ability by augmenting FRA and PON activity. The in silico interaction revealed a substantial binding affinity of moringa’s major phytoconstituents with HMG-CoA reductase. The histological outcomes illustrated the protective effect of moringa against HCD-induced fatty liver changes, hepatic inflammation, and the generation of ROS, as well as cellular senescence in the kidney, ovary, and testes. The study underscores moringa’s potential as a functional food to prevent metabolic stress, organ health, and boost the antioxidant status; however, detailed humanized studies are warranted to substantiate its therapeutic potential.

## Figures and Tables

**Table 1:** Details of the docking between the phytochemicals of *Moringa oleifera* leaf and 3-hydroxy-3-methylglutaryl coenzyme A (HMG-CoA) reductase (PDB ID:1HWK).

Phytoconstituent of *Moringa oleifera* Leaf	Docking Score (kcal/mol)	Residues with H-Bonds	Residues with Hydrophobic Interaction
3-Caffeoylquinic acid (chlorogenic acid)	−10.33	Asp653 (2), Gly656, Asn658 (2), Gly807	Phe628, Ala654, Met655, Met657, Met659, Ala826, Cys827, Pro931,
Quercetin-3-*O*-glucoside (isoquercetin)	−9.45	Ser626 (2), Asn658, Lys662, Asp653 (2)	Phe628, Met659, Ala654, Val805, Ala826, Cys827, Pro831
Kaempferol 3-*O*-rutinoside	−8.51	Asp653, Lys662, Ser626, Gly807, Asn810 (2)	Ala525, Phe628, Ala654, Met659, Ala826, Val805, Cys827, Pro831
Astragalin	−7.76	Ser626, Asp653 (2)	Phe628, Ala654, Met659, Val805, Ala826, Cys827, Pro831
Apigetrin	−7.49	Gly560 (2), Asn658, Gly807	Ala525, Ala654, Phe628, Met655, Met657, Met659, Val805
Myricetin	−7.30	Asp553	Phe628, Ala654, Met655, Met659, Val805, Ala826, Cys827, Pro831

Ala: Alanine; Asp: Aspartic acid; Asn: Asparagine; Gly: glycine; Lys: Lysine; Ser: Serine; Cys: Cysteine; Phe: Phenylalanine; Pro: Proline; Val: Valine; Met: Methionine.

## Data Availability

Data presented in this study is contained within the article and supplementary material. Further inquiries can be directed to the corresponding author.

## Supplementary Materials

The following supporting information can be downloaded at: https://www.mdpi.com/article/10.3390/ph18091336/s1, Supplementary Table S1: Docking score of 25 phytochemical of *M. oleifera* leaf with HMG-CoA reductase. Supplementary Figures S1 and S2: A certificate of analysis of the used moringa leaves. Supplementary Method Section S1. Methodology of the quantification of blood lipoprotein profile hepatic function biomarkers AST and ALT. Supplementary Method Section S2. Methodology for the quantification of plasma paraoxonase and FRA activity.
