# Supplementary material for: Moringa (Moringa oleifera) Leaf Attenuates the High-Cholesterol Diet-Induced Adverse Events in Zebrafish: A 12-Week Dietary Intervention Resulted in an Anti-Obese Effect and Blood Lipid-Lowering Properties
Source: Pharmaceuticals (Basel). 2025 Sep 5;18(9):1336. doi: 10.3390/ph18091336 (PMC12472896; doi:10.3390/ph18091336)
Supplement: Supplementary file 1 [file pharmaceuticals-18-01336-s001.zip › pharmaceuticals-3833256-supplementary.pdf]

Supplementary Material

Supplementary Table S1: Docking score of 24 phytochemical of moringa leaf with HMG-CoA reductase.

S.N	Entry Name	Pub Chem ID	Docking score
1.	3-caffeoylquinic acid (chlorogenic acid)	1794427	−10.33
2.	Quercetin-3-*O*-glucoside (isoquercetin)	5280804	−9.45
3.	kaempferol 3-*O*-rutinoside	5318767	−8.51
4	Astragalin	5282102	−7.76
5.	Apigetrin	5280704	−7.49
6.	Myricetin	5281672	−7.30
7.	Ellagic acid	5281855	−7.19
8.	Quercetin	5280343	−6.49
9.	Kaempferol	5280863	−6.00
10.	Sinapic acid	10743	−5.85
11.	N,α-L-rhamnopyranosyl vincosamide	71717770	−5.69
12.	D (+)-Phenyllactic acid	3848	−5.51
13.	4-[4′-*O*-Acetyl-α-Lrhamnosyloxy) benzyl] Isothiocyanate	10291650	−5.48
14.	Moringin	153557	−5.26
15.	Niazirin	129556	−5.23
16.	Sinalbin	76956748	−5.05
17.	4-Undecylbenzenesulfonic acid	38222	−4.71
18.	β-sitosterol	222284	−4.48
19.	Caffeic acid	689043	−4.40
20.	Syringic acid	10742	−4.33
21.	Salicylic acid	338	−4.18
22.	4-Hydroxybenzaldehyde	126	−4.07
23.	Ferulic acid	445858	−3.41
24.	Marumosid B (4′-hydroxyphenylethanamide)	86986	−3.27
25.	Methyl-p-hydroxybenzoate (methyl paraben)	7456	−3.19

Supplementary Figure S1: A certificate of analysis of the used moringa leaves.

한국식품과학연구원
Korea Advanced Food Research Institute

Korea Advanced Food Research Institute

50, Botdeul-ro, Uiwang-si, Gyeonggi-do, Republic of Korea
TEL : 82-2-3470-8200 FAX : 82-2-523-2072 https://www.kafri.or.kr

KAFRI

Certificate of Laboratory Testing(Reference)

Receipt No.	2023-11-013088	Date of Receipt	2023.08.02
Product Name	Moringa Powder MPV21027		
Client Company Name	RAYDEL KOREA CO.,LTD		
Client Address	No. 205, 2nd Fl, 15-10, Gangnam-daero 39-gil, Seocho-gu, Seoul, Republic of Korea		
Client Name	LEE BYONG KU	Batch Number	MPV21027
Date of Manufacturing (Import)	2021.10.14	Expiration(Quality Assurance) Date	2023.10.13
Test Purpose	For confirmation(in company)	Date of Issue	2023.08.09

Test Items and Results

Dehydroacetic acid(g/kg)..........................Not Detected
Sorbic acid(g/kg)................................Not Detected
Benzoic acid(g/kg)...............................Not Detected
p-Hydroxybenzoate(g/kg)..........................Not Detected
Propionic acid(g/kg).............................Not Detected
Foreign materials(metallicity)(mg/kg).............0.4
Sulfur dioxide(g/kg).............................Not Detected
Escherichia coli.................................0/g,0/g,0/g,0/g,0/g

* The above test items and results complied with the test method notified by MFDS.

Testing Jiyoung Moon
Testing Manager Eunhee Kim

Note 1. The above merchandise was submitted and identified by the client.
2. The results shown in this report refer only to sample tested and it does not cover the quality of all products.
3. No one can use this report for the purpose of test, advertisement and litigation without KAFRI's consent.
4. This report has no legal effect and authenticity can be checked through the QR code located in the lower left corner.
5. This report is not related to KS Q ISO/IEC 17025 and KOLAS Accreditation.

This certificate does not comply with the Act on Testing and inspection of Food and Drugs of Ministry of Food and Drug Safe.

Authenticity check

The President of Korea Advanced Food Research Institute

COPY 복사 VOID

Supplementary Figure S2: A certificate of analysis for the quantification of Kaempferol in the moringa leaves powder.

English translated

Koptri

Korea Polymer Testing & Research Institute

www.polymer.co.kr

Test report No.: 22-02313

TEST REPORT

1. Requesting organization:

Organization Name: Raydel Korea (Chang-Hwan Lee)

[illegible] #205 Hanla Vivaldi, 15-10 Gangnam-daero 39-gil, Seocho-gu, Seoul

2. Date of request: March 3, 2022

3. Purpose: For research purposes

4. Sample shape: solid

5. Test items: See Table 2

6. Test method: See Table 2.

7. Exam Period: March 10, 2022 - March 29, 2022

8. Test results: See Table 2

9. Tester: Senior Researcher Kim Mi-kyung

check	hands-on worker<br>Name: Kim Mi-kyung	Mikyoung (young) kim	Technical Manager<br>Name: Park Ji-young	

This test report is the measurement result using the sample provided by the client.

This Test Report cannot be used for propaganda, broadcasting, advertising, or legal action without prior consent, and copying and use are prohibited.

This test report is not related to KS Q ISO/IEC 17025 and KOLAS accreditation.

March 29, 2022

Korea Polymer Testing & Research Institute, an internationally accredited testing institute (인) Heeyeon

English translated

Koptri
Korea Polymer Testing & Research Institute

TEST REPORT

Sample name	item	unit	method	Detection limit	result
Koptri-22-07-01951	Kaempferol	mg/kg	LC-MS/MS	0.001	7.7

Note a) mg/kg = ppm

b) LC-MS/MS ; Liquid Chromatography tandem Mass Spectrometry

c) Koptri-NB-VI20-SW01.0-EA-MOH10

end.

Supplementary Method

Section S1. Quantification of blood lipoprotein profile hepatic function biomarkers AST and ALT

The plasma total cholesterol (TC) and triglycerides (TGs) were determined using commercial assay kits (cholesterol, T-CHO, and TGs, Cleantech TS-S; Wako Pure Chemical, Osaka, Japan) as per the method suggested by the suppliers. In brief, 5 μL serum was mixed with 200 μL reaction mixture (supplied with a commercial assay kit) for the TC analysis. The content was incubated at 37 °C for 10 min, resulting in a red-colored product quantified by adsorption at 490 nm (Microplate reader, Bio-Rad, Hercules, CA, USA).

Similarly, 5 μL serum was mixed with a 200 μL of TGs-specific reaction mixture (supplied with a commercial assay kit) for TGs analysis. The content was incubated for 10 min at 37 °C, and the formed colored product was quantified by taking adsorption at 490 nm.

For HDL-C analysis, serum was mixed in an equal ratio with the separation solution (supplied with a commercial assay kit), followed by centrifugation at 3000 rpm for 10 min. The supernatant (20 μL) was collected and blended with a 200 μL reaction mixture (supplied with a commercial assay kit). After 10 min incubation at 37°C, red color intensity corresponding to HDL-C was quantified by taking absorption at 490 nm.

The LDL-C level was quantified using the Friedewald equation:

$$LDL\text{-}C = TC - HDL\text{-}C - (TG/5)$$

The non-HDL-C level was quantified using the:

$$Non\text{-}HDL\text{-}C = TC - HDL\text{-}C$$

The atherogenic coefficient and atherogenic index were calculated using the equations:

(non-HDL-C/HDL-C) and log (TG/HDL-C), respectively.

The commercial diagnostic kit (Asan Pharmaceutical, Hwasung, Republic of Korea) was used to quantify aspartate transaminase (AST) and alanine transaminase (ALT) levels in the plasma, following the instructions suggested by the manufacturers. Briefly, 5 μL of plasma was combined with 250 μL of either AST or ALT-specific solution, as supplied in the diagnostic kit. Following a 30 min incubation for AST or 60 min incubation of ALT at 37°C, the mixture was then blended with 250 μL of the respective coloring reagent (AST or ATL-specific, provided in the diagnostic kit). After a subsequent 20 min incubation at room temperature, 250 μL of 0.4 N NaOH was introduced to halt the reaction. Finally, the AST and ATL were quantified by measuring absorbance at 490 nm.

Section S2. Paraoxonase and FRA activity

Briefly, 40 μL of the plasma (1 mg mL^{-1} equivalent protein) was suspended in the 180 μL of paraoxon ethyl (0.15 g mL^{-1}) and content was incubated at RT for 120 min. The absorbance 415 nm was determined and the paraoxonase activity was expressed as μU L^{-1} min^{-1} using the molar absorbance coefficient ($\varepsilon = 17 \times 10^3$ M^{-1} cm^{-1}) of p-nitrophenol, a product formed by the action of paraoxonase.

To assess ferric ion reduction (FRA) capacity, 20 μL of the plasma (1 mg mL^{-1} equivalent protein) was mixed with 180 μL of FRA reagent (prepared by blending 10 mL of acetate buffer (0.2 M, pH 3.6) with 1.25 mL each of 2,4,6-tripridyl-S triazin (10 mM) and $FeCl_3$ (20 mM). After incubating the mixture at RT for 60 min, absorbance was measured at 593 nm. The results were quantified in μM ferric equivalents based on a ferrous sulfate standard curve.
